# Completion of the sequence of the Aspergillus fumigatus partitivirus 1 genome

**DOI:** 10.1007/s00705-020-04660-0

**Published:** 2020-05-27

**Authors:** Charalampos Filippou, Robert H. A. Coutts, David A. Stevens, Raquel Sabino, Ioly Kotta-Loizou

**Affiliations:** 1 Department of Biological and Environmental Sciences, School of Life and Medical Sciences, University of Hertfordshire, Hatfield, UK; 2 Division of Infectious Diseases and Geographic Medicine, Stanford University School of Medicine, Stanford, California USA; 3 California Institute for Medical Research, San Jose, California USA; 4 Infectious Diseases Department, National Institute of Health Dr. Ricardo Jorge, Lisbon, Portugal; 5 Department of Life Sciences, Faculty of Natural Sciences, Imperial College London, London, UK

## Abstract

A Portuguese isolate of *Aspergillus fumigatus* was found to contain three double-stranded (ds) RNA elements ranging in size from 1.1 to 1.8 kbp and comprising the genome of a strain of Aspergillus fumigatus partitivirus 1 (AfuPV-1) previously thought to contain only the two largest dsRNA elements. The sequence of the smallest dsRNA element is described here, completing the sequence of the AfuPV-1 genome. Sequence analysis of the element revealed an open reading frame encoding a protein of unknown function similar in size and distantly related to elements previously identified in other members of the family *Partitiviridae*.

Mycoviruses are widespread in almost all major groups of fungi, and most cause no obvious effects in their hosts, but some do cause obvious symptoms resulting in debilitated virulence, slow growth rate, and poor sporulation [5, 8]. Currently recognized mycoviruses have single-stranded (ss) or double-stranded (ds) RNA, or rarely DNA, as their genetic material [8]. Members of one family, the *Partitiviridae,* are classified into five genera, namely *Alphapartitiviru*s, *Betapartitivirus*, *Gammapartitivirus*, *Deltapartitivirus* and *Cryspovirus* [17]. Partitiviruses generally possess two essential dsRNA genome segments (1.3-2.5 kbp in length), each containing a single open reading frame (ORF) [17]. The larger component 1 encodes an RNA-dependent RNA polymerase (RdRP), and the smaller component 2 encodes the capsid protein (CP) [17]. On occasion, smaller dsRNAs than the two dsRNAs described above encoding proteins of unknown function have been reported for some partitiviruses. Examples include Ustilaginoidea virens partitivirus 1 (UvPV-1) [18], Gremmeniella abietina RNA viruses MS1, MS2 and MS3 (GaRV-MS1, -MS2 and -MS3) [3, 15, 16], Aspergillus ochraceous virus (AoV) [10, 11] and Aspergillus flavus partitivirus 1 (AfPV-1) [6], but not Aspergillus fumigatus partitivirus 1 (AfuPV-1) [2] or a second isolate of UvPV [7]. During our continued monitoring of the incidence of mycoviruses in *Aspergillus fumigatus*, an opportunistic pathogen causing aspergillosis in the immunocompromised human population, we isolated, cloned and sequenced a Portuguese strain of AfuPV-1 termed AfuPV-1A. An examination of the dsRNA profile of AfuPV-1A revealed that it contained three dsRNA components, with the sequences of the two largest dsRNAs being 98-99% identical to those of AfuPV-1. These results confirmed that AfuPV-1A is a strain of AfuPV-1 that can support the replication of a third genomic component termed dsRNA3 and that smaller dsRNA components can apparently be lost and recovered during partitivirus infections. Here, the sequence of the AfuPV-1A dsRNA3 element is reported, completing the sequence of the AfuPV-1A genome. Additionally, relationships between AfuPV-1A dsRNA3 and AfuPV-1 dsRNAs 1 and 2 were investigated together with comparisons to other small dsRNA elements of partitiviruses.

## Provenance of the virus material

**Fig. 1:**
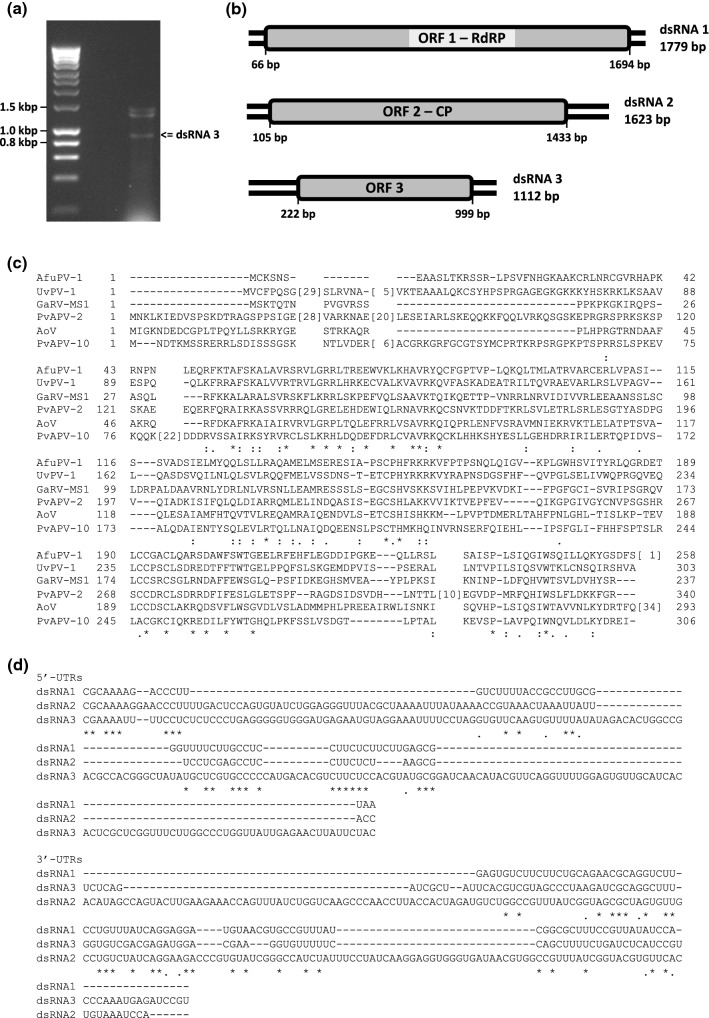
Genomic organisation of AfuPV-1 and comparisons of the amino acid sequences of putative proteins encoded by dsRNA3 elements of representative partitiviruses. **(a)** Agarose gel electrophoresis of dsRNA elements extracted from *A. fumigatus* isolate 12/43 Molecular sizes are indicated on the left with numbers. **(b)** Schematic representation of the genome organisation of AfuPV-1A. AfuPV-1 dsRNAs 1, 2 and 3 each contain a single ORF (grey boxes) flanked by 5’- and 3’-UTRs (black lines). The dsRNA1 is 1779 bp long and contains an ORF that encodes a putative RdRp with a molecular mass (*Mr*) of 63 kDa. The dsRNA2 is 1623 bp long and contains an ORF that encodes a putative CP with a *Mr* of 48 kDa. The dsRNA3 is 1112 bp long and contains an ORF that encodes a protein of unknown function with a *Mr* of 29 kDa. **(c)** BLAST COBALT alignment of the amino acid sequences of the putative proteins of unknown function encoded by the smallest genomic dsRNA3 elements of Aspergillus fumigatus partitivirus-1A (AfuPV-1A; LR743535), Ustilaginoidea virens partitivirus 1 (UvPV-1; AGO04405), Gremmeniella abietina RNA virus-MS1 (GaRV-MS1; AII16003), Plasmopara viticola associated partitivirus 2 (PvAPV-2; QHD64806), Aspergillus ochraceous virus (AoV; AYP71820) and Plasmopara viticola associated partitivirus 10 (PvAPV-10; QHD64798). Accession numbers are shown in brackets above. In the sequence alignment, asterisks signify identical amino acid residues, colons signify highly conserved residues, and single dots signify less-conserved but related residues. **(d)** Alignment of the 5’- and 3’-UTRs of the coding strands of the three genomic segments of AfuPV-1A, where asterisks indicate identical nucleotides in the three elements and single dots signify conserved purines or pyrimidines

*A. fumigatus* 12-43 was originally isolated from bronchial secretions of a hospitalised patient in Portugal [12]. This was the only *A. fumigatus* isolate found to harbor dsRNA elements following the screening of 48 isolates in total: 12 environmental, 12 avian, 12 from cystic fibrosis patients, and 12, including isolate 12-43, from non-cystic fibrosis patients [12–14]. The *A. fumigatus* isolate 12-43 was grown in liquid Czapek-Dox complete medium at 25 °C with shaking. Purification of virus particles was performed as described previously [9], and dsRNA was extracted from the particles using phenol/Sevag treatment. Three viral dsRNA elements were separated by electrophoresis on a 1% (w/v) agarose gel containing Tris-acetate-EDTA (TAE) buffer and 500 ng of ethidium bromide per ml (Fig. 1a). The two larger dsRNAs 1 and 2 of the *A. fumigatus* isolate 12-43 were 1779 and 1623 bp in size, respectively, and were 99% and 98% identical in sequence to those previously reported to constitute the genome of AfuPV-1 [2]. The additional smaller dsRNA is referred to as “AfuPV-1A dsRNA3” in this study. AfuPV-1A dsRNA3 was extracted from the gel using a MinElute Gel Extraction Kit (QIAGEN) and its dsRNA nature was confirmed by its resistance to DNase 1 and S1 nuclease (Promega). Subsequently, the dsRNA was denatured with methyl mercuric hydroxide, and this served as a template for reverse transcription and PCR amplification using random primers [5]. The sequence was completed using sequence specific primers, genome walking, and RNA ligase-mediated rapid amplification of cDNA ends [4]. All products were cloned using the pGEM-T Easy Vector system (Promega) and introduced into competent *Escherichia coli* XL10-Gold cells (Agilent). At least three different clones covering the same part of the genome were sequenced. The effects of AfuPV-1A on the phenotype and pathogenicity of its host are currently unknown but are assumed to be similar to those described previously by Bhatti *et al*. [2] who observed an abnormal colony phenotype, slow growth, and lighter-than-normal pigmentation.

## Sequence properties

AfuPV-1A dsRNA3 is 1112 bp in length, has 50% GC content and possesses a single ORF that encodes a hypothetical protein of unknown function comprising 258 amino acids (aa) with a molecular mass of 29.274 kDa (Fig. 1b). A BLASTp [1] search using the non-redundant protein sequences database updated on 6 April 2020 revealed that the amino acid sequence of the protein encoded by the AfuPV-1A dsRNA3 ORF was 41.5%, 32.6% and 31.5% identical to that of the corresponding protein of unknown function encoded by the smallest genomic dsRNA3 elements of UvPV-1 (303 aa), AoV (293 aa) and GaRV-MS1 (237 aa), respectively. It also demonstrated 33.2% and 30.3% sequence identity to dsRNA3 of the recently reported Plasmopara viticola associated partitivirus 2 (PvAPV-2; 340 aa) and Plasmopara viticola associated partitivirus 10 (PvAPV-10; 306 aa), respectively (Fig. 1c). Neither GaRV-MS2 nor GaRV-MS3 was included in these alignments, as they are considered to be strains of GaRV-MS1 [3, 16]. The AfuPV-1A dsRNA3 ORF is flanked by 5’- and 3’-UTRs 222 and 113 bp in length, respectively. A comparison between the 5’-UTRs of AfuPV-1A dsRNAs 1, 2 and 3 illustrated that they differed in length but showed some conserved nucleotides at the 5’ terminus (CGC/AAAAA/UG/U; Fig. 1d). This is consistent with observations for multi-component RNA viruses, where the 5’-terminal sequences are essential for recognition by the viral RdRP during viral RNA replication [6]. Such conserved sequences proximal to the 5’termini of partitiviruses are thought to be involved in RdRP recognition for RNA packaging and/or replication [17]. The 3’-UTRs differ in length, and there is little sequence conservation (Fig. 1d). *In silico* analysis of the entire sequence of AfuPV-1A dsRNA3, including both UTRs, revealed the presence of stem-loop and panhandle structures (results not shown), but their significance in replication is unknown [17]. It is not known why some members of the family *Partitiviridae* possess a bipartite genome while others appear to be tripartite, as is the case now for AfuPV-1A. It is curious that the smallest dsRNA element was not isolated in our initial screens of dsRNA elements in *A. fumigatus*, but the appearance and disappearance of mycovirus elements in some instances is thought to be related to fungal growth conditions and variable virus distribution in mycelia. In the absence of reverse genetics to investigate whether AfuPV-1A dsRNA3 and similar dsRNAs in other partitiviruses are essential, any possible roles in replication remain unknown.

## References

[1] Altschul SF, Madden TL, Schaffer AA, Zhang J, Zhang Z, Miller W, Lipman DJ (1997). Gapped BLAST and PSI-BLAST: a new generation of protein database search programs. Nucleic Acids Res.

[2] Bhatti MF, Bignell EM, Coutts RHA (2011). Complete nucleotide sequences of two dsRNAs associated with a new partitivirus infecting *Aspergillus fumigatus*. Arch Virol.

[3] Botella L, Tuomivirta TT, Hantula J, Diez JJ, Jankovsky L (2015). The European race of *Gremmeniella abietina* hosts a single species of *Gammapartitivirus* showing a global distribution and possible recombinant events in its history. Fungal Biol.

[4] Coutts RHA, Livieratos IC (2003). A rapid method for sequencing the 5’- and 3’-termini of dsRNA viral templates using RLM RACE. J Phytopathol.

[5] Froussard P (1992). A random-PCR method (rPCR) to construct whole cDNA library from low amounts of RNA. Nucleic Acids Res.

[6] Jiang Y, Wang J, Yang B, Wang Q, Zhou J, Yu W (2019) Molecular characterization of a debilitation-associated partitivirus Infecting the pathogenic fungus *Aspergillus flavus* (2019). Front Microbiol. 10.3389/fmicb.2019.00626 10.3389/fmicb.2019.00626 PMC6447663 30984147

[7] Jiang Y, Luo C, Jiang D, Li G, Huang J (2014). The complete genomic sequence of a second novel partitivirus infecting *Ustilaginoidea virens*. Arch Virol.

[8] Kotta-Loizou I, Coutts RHA (2017). Mycoviruses in *Aspergilli*: a comprehensive review. Front Microbiol.

[9] Kotta-Loizou I, Coutts RHA (2017). Studies on the virome of the entomopathogenic fungus *Beauveria bassiana* reveal novel dsRNA elements and mild hypervirulence. PLoS Pathog.

[10] Liu W, Duns G, Chen J (2005). Genomic characterization of a novel partitivirus infecting *Aspergillus ochraceus*. Virus Genes.

[11] Nerva L, Forgia M, Ciuffo M, Chitarra W, Chiapello M, Vallino M, Varese GC, Turina M (2019). The mycovirome of a fungal collection from the sea cucumber *Holothuria polii*. Virus Res.

[12] Sabino R, Veríssimo C, Parada H, Brandão J, Viegas C, Carolino E, Clemons KV, Stevens DA (2014). Molecular screening of 246 Portuguese *Aspergillus* isolates among different clinical and environmental sources. Med Mycol.

[13] Sabino R, Ferreira JA, Moss RB, Valente J, Veríssimo C, Carolino E, Clemons KV, Everson C, Banaei N, Penner J, Stevens DA (2015). Molecular epidemiology of *Aspergillus* collected from cystic fibrosis patients. J Cyst Fibros.

[14] Sabino R, Burco J, Valente J, Veríssimo C, Clemons KV, Stevens DA, Tell LA (2019). Molecular identification of clinical and environmental avian *Aspergillus* isolates. Arch Microbiol.

[15] Tuomivirta TT, Hantula J (2003). Two unrelated double-stranded RNA molecule patterns in *Gremmeniella abietina* type A code for putative viruses of the families *Totiviridae* and *Partitiviridae*. Arch Virol.

[16] Tuomivirta TT, Hantula J (2005). Three unrelated viruses occur in a single isolate of *Gremmeniella abietina* var. abietina type A. Virus Res.

[17] Vainio EJ, Chiba S, Ghabrial SA, Maiss E, Roossinck M, Sabanadzovic S, Suzuki N, Xie J, Nibert M (2018). ICTV virus taxonomy profile: *Partitiviridae*. J Gen Virol.

[18] Zhang T, Jiang Y, Huang J, Dong W (2013). Genomic organization of a novel partitivirus from the phytopathogenic fungus *Ustilaginoidea virens*. Arch Virol.

